# Microbiological and Physicochemical Characteristics of Three Types of “Soumbara” from Seeds of African Locust Bean in Korhogo Markets, Côte d'Ivoire

**DOI:** 10.1155/2021/5572300

**Published:** 2021-04-10

**Authors:** Ollo Kambire, Konan Mathurin Yao, Andrée Emmanuelle Sika, Aminata Coulibaly, Zamble Bi Irie Abel Boli, Rose Koffi-Nevry

**Affiliations:** ^1^Department of Biochemistry and Genetics, Peleforo Gon Coulibaly University, BP 1328 Korhogo, Côte d'Ivoire; ^2^Department of Food Science and Technology, Laboratory of Biotechnology and Food Microbiology, Nangui Abrogoua University, 02 BP 801 Abidjan, Côte d'Ivoire

## Abstract

“Soumbara” is a fermented product sold in the markets of several West African countries. In the markets, it is sold in several formats (granulated, powder, and paste). The objective of this study was to evaluate the microbiological and physicochemical characteristics of these three types of “Soumbara” sold in the Korhogo markets. For this purpose, a preliminary survey followed by a sampling of 54 samples of “Soumbara” was carried out. The microorganism load count was carried out according to microbiological standards. The pH, titratable acidity, and moisture content were measured, respectively, with a pH meter, by dosing with sodium hydroxide solution and by differential weighing after passing the sample through the oven. The pH of the different samples is around 6. The moisture content is higher in “Soumbara” paste (20-24.7%) than in powdered (7.3-9.3%) and granulated (8.6-10.7%) “Soumbara.” The acidity rates are between 0.07 and 0.13%, 0.2 and 0.3%, and 0.08 and 0.1%, respectively, for the granulated, powder, and paste types. Mesophilic aerobic germ loads (6.17-8.38 log_10_ cfu/g) for all three types of “Soumbara” are above the standard. Total coliform (1.13-2.96 log_10_ cfu/g), mould (0.86-2.52 log_10_ cfu/g), and yeast (0.33-1.53 log_10_ cfu/g) loads are below standard. The microbiological quality of the three types of “Soumbara” is unsatisfactory. Overall, “Soumbara” powder is the most contaminated, followed by granulated and paste “Soumbara.” “Soumbara” must be added during culinary preparations in order to avoid possible public health problems.

## 1. Introduction

Food is a fundamental need for life. Agriculture, animal husbandry, and fishing provide humans with a variety of products that must be preserved over a long period of time. However, there are great disparities in food availability, particularly in sub-Saharan Africa where food insecurity, galloping population growth, and unbridled urbanization make people fear the worst for the future [1]. To this end, rapid urbanization in West Africa is contributing to the development of a processing and marketing sector for local agricultural products, essentially artisanal [2]. During the processing of these local products, a process of natural fermentation of protein and oil seeds takes place, particularly those of baobab (*Adansonia digitata*), groundnut (*Arachis hypogaea*), *Hibiscus sabdariffa*, soybean (*Glycine max*) and an African locust bean (*Parkia biglobosa*) [3, 4]. The fermentation of seeds of the *Parkia biglobosa* species produces a condiment that is one of the most important resources for food security, especially in times of food shortage and drought, and a resource of important socioeconomic and cultural values for local populations [5]. The fermentation of *Parkia biglobosa* seeds carried out by the *Bacillus* genus is an alkaline fermentation. This condiment is known in West Africa as “Soumbara” in Côte d'Ivoire, “soumbala” in Burkina Faso and Mali, “dawa-dawa” in Niger and Nigeria, and “nététu” in Benin and Senegal [6–9]. In most West African countries, “soumbala” is widely consumed by the rural and urban population. It has been accepted by public opinion that this condiment has a potential ability to regulate blood pressure, creating a growing craze for this condiment in cities [4, 10].

Produced however in an artisanal method and sold in the different markets in unhygienic conditions, the “Soumbara” could harbour microorganisms that can be pathogenic for humans and degrade the nutritional quality of this product. The “Soumbara” provides a favourable environment for the development of microorganisms due to its high water and nutrient content. Soumbara can be eaten after cooking in sauces. It can also be used to season dishes without cooking (especially powdered Soumbara). This act could create a health problem for the consumer if this condiment contains pathogens.

In Côte d'Ivoire, the main centres of production of “Soumbara” are located in the far north of the country where the resources of *Parkia biglobosa* are abundant. The production activities are still artisanal and devolved to women. According to De Angelis et al. [11], it is important to maintain local traditional foods which are often more environmentally sustainable than imported ones and have also a cultural meaning.

This study was undertaken in order to evaluate the physicochemical and microbiological characteristics of three types of “Soumbara” sold in different markets in the town of Korhogo.

## 2. Material and Methods

### 2.1. Survey

A preliminary survey was carried out among the “Soumbara” sellers in the different markets (five markets). This survey was aimed to collect information on the different types of “Soumbara” sold in the markets, the most sold type, the place of supply, the method of conservation, and hygiene. This survey was conducted in Korhogo, a city in the north of Côte d'Ivoire.

### 2.2. Sample Collection

**Figure 1:**
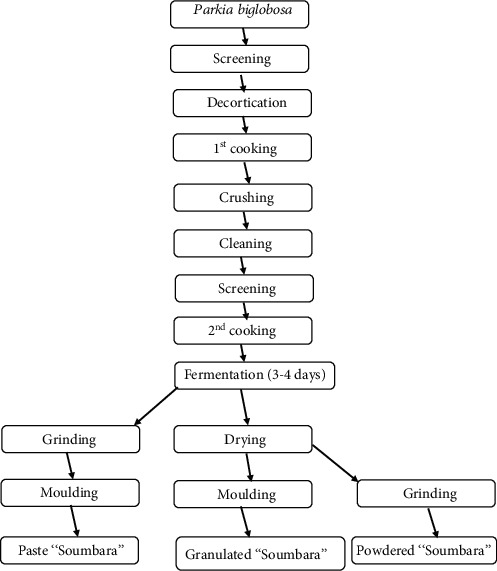
Diagram of the different production steps of the three types of “Soumbara”.

The “Soumbara” production diagram contains several steps (Figure 1). In the different markets, “Soumbara” is sold unpackaged.

Samples were collected in three markets in the city of Korhogo, namely, Haoussabougou market (MH), Petit Paris market (MPP), and Major market (MG) during the period from July to August 2020. The choice of these three markets is based on the effective presence of the different types of “Soumbara” (granulated, powder, paste). For the sampling, three (03) sellers were chosen at random in each market. Two samples of each type of “Soumbara” of approximately 100 g each were taken from each seller, making a total of 54 samples taken. All samples were taken in stomacher bags and transported to the laboratory in a cooler for analysis.

### 2.3. Physicochemical Analysis

#### 2.3.1. pH

The pH was determined according to the method proposed by N'goran-aw et al. [12]. Twenty (20) grams of each sample of “Soumbara” was added to 100 mL of distilled water. After filtration, the pH was read using a HANNA pH meter.

#### 2.3.2. Titratable Acidity

The titratable acidity was determined according to the method described by Amoa-awua et al. [13]. Ten grams of “Soumbara” was homogenized in 100 mL of distilled water. After filtration, 2 to 3 drops of 1% phenolphthalein were added to 10 mL of filtrate taken for the test sample, and the mixture was dosed with 0.1 N sodium hydroxide solution. The end of the dosage was marked by a pale pink colouring. The acidity rate expressed as a percentage was obtained according to the following formula:

(1) $$\begin{array}{c} \%\text{titratable acidity}=\frac{\text{V}\left(\text{NaOH}\right)\text{x N}\left(\text{NaOH}\right)\text{ x }0,09\text{ x }100}{\text{V }\left(\text{test sample}\right)}. \end{array}$$

#### 2.3.3. Moisture Content

The moisture content was determined by differential weighing after oven drying according to the AOAC method [14]. Five (5) grams of each sample was weighed (M1) and placed in a crucible. The crucible containing the sample (M2) was placed in the oven at 105°C for 24 hours and weighed after cooling in a dry place. This process is repeated at one-hour intervals several times until a constant mass (M3) was reached. The moisture content expressed as a percentage was determined according to the following formula:

(2) $$\begin{array}{c} \text{MC}=\frac{M2\;\left(g\right)-M3\left(g\right)}{M1\;\left(g\right)}\times 100. \end{array}$$

### 2.4. Microbiological Analysis

Sample preparation: ten (10) grams of each sample of “Soumbara” was taken aseptically and homogenized in a borosilicate bottle containing 90 mL of buffered peptone solution. From the suspension obtained, decimal dilutions have been performed. Mesophilic aerobic germ count was performed on Plate Count Agar. The inoculated Petri dishes were incubated at 37°C/24 hours. The total coliforms count was carried out on the VRBL. Incubation of the Petri dishes was carried out at 35°C for 24 hours. Dichloran Rose Bengal Chloramphenicol agar was used for the enumeration of yeasts and moulds. The different microbial loads expressed in cfu/g have been calculated according to the formula ISO 7218 [15]:

(3) $$\begin{array}{c} \text{N}\left(\text{cfu}/\text{g}\right)=\frac{\Sigma \text{C}}{\left(\text{n}1+0,1\text{n}2\right).\text{d}.\text{V}}. \end{array}$$

### 2.5. Statistical Analysis

The analysis of variance (one-factor ANOVA) was performed with the Statistica software version 7.1 at the significance level (*α* = 0.05). In case of significant difference between the parameters studied, the ranking of the means is done according to the Newman-Keuls test.

## 3. Results

### 3.1. Survey

In total, three types of “Soumbara” have been identified. These three types are sold simultaneously in three of the five markets surveyed. These are major market, Haoussabougou market and Petit paris market. Only two types are sold in the Soba and Sinistré markets. The type of “Soumbara” most requested by consumers is powdered “Soumbara” (62.4%) followed by granulated “Soumbara” (34.72%). Soumbara paste is the least requested (2.86%). All the saleswomen buy their products from the producers and take more than a day to sell their stock. At all the saleswomen, the unsold “Soumbara” is stored in the store. During marketing, the “Soumbara” is not protected by any of the saleswomen surveyed (Table 1).

### 3.2. pH

The averages obtained ranged from 6.05 to 6.8. The lowest averages were obtained in “Soumbara” powder, but compared to the averages of the other two types (paste, granulated), there is no significant difference (*P* > 0.05) (Table 2).

### 3.3. Titratable Acidity

The average of titratable acidity of the samples of granulated “Soumbara” (0.07-0.13%) is close to that of “Soumbara” paste (0.08-0.1%). The highest percentages of titratable acidity were recorded in the samples of “Soumbara” powder (0.2-0.3%). However, apart from the acidity of the samples of “Soumbara” powder from the major market, which stands out significantly (*P* < 0.05), no significant difference was recorded between the other titratable acidity values (Table 3).

### 3.4. Moisture Content

The moisture content obtained was between 7.3 and 24.7%. The most important moisture contents (*P* < 0.05) were recorded in the samples of “Soumbara” paste. The lowest rate (7.3%) was measured in samples of “Soumbara” powder. There is no significant difference between the values of granulated “Soumbara” and “Soumbara” powder (Table 4).

### 3.5. Microbiological Analysis

#### 3.5.1. Mesophilic Aerobic Germ

Mesophilic aerobic germ loads in the different types of “Soumbara” range from 6.17 to 8.38 log_10_ cfu/g. They were between 6.41 and 8.38 log_10_ cfu/g for granulated “Soumbara,” 6.94 and 7.74 log_10_ cfu/g for powdered “Soumbara” and between 6.17 and 8.74 log_10_ cfu/g for paste “Soumbara.” The majority of the significant charges were recorded in the granulated type. The loads of the three “Soumbara” types obtained in the samples of the major market were higher (*P* > 0.05) than those of the other two markets. There was no significant difference (*P* > 0.05) in loads from one type to another. All loads were above the limit (*M*) value of 6 log_10_ cfu/g (Table 5).

#### 3.5.2. Total Coliforms

The loads were between 1.13 and 2.96 log_10_ cfu/g for all types. For each type of “Soumbara,” the loads were between 1.71 and 2.25, 1.86 and 2.96, and 1.13 and 1.64 log_10_ cfu/g for the granulated, powdered, and paste types, respectively. The most important loads were obtained in the powdered type; however, there is no significant difference between these loads and those of the other two “Soumbara” types. For all types of “Soumbara,” the loads are lower than the limit (*M*), which was 3 log_10_ cfu/g (Table 6).

#### 3.5.3. Yeasts and Moulds

The mould loads of the different types of “Soumbara” were between 0.86 and 2.52 log_10_ cfu/g. For each type, the loads were between 1.44 and 2.4, 1.76 and 2.25, and 0.86 and 1.56 log_10_ cfu/g for the granulated, powdered, and paste types, respectively. The powder type contains higher loads, but there were no significant differences (*P* > 0.05) between these and the other two types. All mould loads were below the limit (*M*) value (4 log_10_ cfu/g) (Table 7).

No yeast was counted in the three types of “Soumbara” of major market. For the other two markets, loads are between 0.33 and 1.53 log_10_ cfu/g. The samples of “Soumbara” powdered recorded the highest loads, but these were not significantly different from the other types. All yeast loads were below the value of the limit (*M*) (4 log_10_ cfu/g) (Table 8).

## 4. Discussion

The range of pH obtained for the three types of “Soumbara” is between 6.05 and 6.8, and there is no significant difference from one type to another. These values are within the pH range obtained by Kambire et al. [16] in the “Soumbara” sold in the markets of the city of Abidjan. According to these authors, the acid pH obtained in the different samples of “Soumbara” is linked to the fermentation time (short). Indeed, for Parkouda et al. [17], the fermentation of African locust bean seeds is an alkaline fermentation. Thus, the low titratable acidity values obtained in this study could justify this predominance of alkaline fermentation.

The moisture content obtained in the samples of “Soumbara” in paste form are significantly higher than those of the other two types (granulated and powdered). This important difference in moisture content is linked to the production process of “Soumbara.” Indeed, a drying stage is necessary during production to obtain granulated and powdered “Soumbara” unlike paste “Soumbara.” The moisture content of the “Soumbara” paste is within the range of the moisture content values reported (15-27%) by Camara et al. [18] in the same product. The water content of food products plays a decisive role during their storage. This is a parameter that significantly affects the conservation and development time of microbial contaminants [19].

In the different markets, the “Soumbara” is sold without any protection. This condiment is sold unpackaged; moreover, the conservation of the unsold “Soumbara” is done in the stores where the temperature of conservation is not respected. All these bad practices could influence the microbiological contamination level of this condiment in the markets.

All mesophilic aerobic germ loads for the three types of “Soumbara” in this study are higher than the guide value of 6 log_10_ cfu/g, indicating an unsatisfactory microbiological quality of the different samples. Lack of personnel hygiene, storage in improper conditions, and prolongation of the storage period due to delay in its sale could be at the origin of these high loads. This result corroborates that of Kambire et al. [16]. The variance of loads between the three types of “Soumbara” is not significant. However, it must be stressed that the mesophilic aerobic germ loads for the three types of “Soumbara” of the major market are the most important. These important loads recorded in this market would be due to the high traffic in this market compared to the other two. According to Kasse et al. [20], the variability in mesophilic aerobic germ from one vendor to another could depend on the density of street traffic, which influences environmental hygiene and therefore product contamination. High loads of mesophilic aerobic germs would favour a strong alteration of the product (merchantable quality) thus generating economic losses.

The presence of total coliforms in the different types of “Soumbara” would be due to a lack of hygiene during production and marketing, but also to the conditions of conservation. Total coliform loads are almost identical to those reported by Somda et al. [21] but are higher than the results of Parkouda et al. [17]. However, they are lower than the guide value of 3 log_10_ cfu/g.

Mould loads are higher than yeast loads but lower than the guide value (4 log_10_ cfu/g). This result differs from that of Degnon et al. [22], who found no mould loads in the “afitin” a condiment similar to “Soumbara.” Fungi are ubiquitous microorganisms that can grow on a wide variety of substrates. Contamination could take place during the processing of African locust bean seeds, marketing, and storage. Moulds are capable of producing mycotoxins in food products. Dabire et al. [10] detected the presence of aflatoxin B2 in samples of “Soumbara” sold in Burkina Faso.

Overall, the microorganism loads of powdered “Soumbara” form are the highest, followed by the loads of granulated “Soumbara” form and finally paste “Soumbara” form despite its higher moisture content than the other two. The level of contamination would be more related to daily contamination and to the format of the “Soumbara.” Indeed, the “Soumbara” powder has a large contact surface for contamination and is easily mixed compared to the other two.

## 5. Conclusion

The objective of this study was to carry out a microbiological and physicochemical characterisation of three formats of “Soumbara” sold in the markets of Korhogo. The results of this study show that the microbiological quality of the three formats of “Soumbara” sold in Korhogo is unsatisfactory. Mesophilic aerobic germs are responsible for this unsatisfactory result. These microorganisms could degrade this condiment by altering the taste, the smell, and the aspect, in sum the merchantability of the “Soumbara.” Overall, the level of contamination is higher in “Soumbara” powder. This format is most often used in food without any cooking. The consumer must add the “Soumbara” during the cooking of the meals. Producers and sellers should adopt adequate hygienic conditions to preserve the safety of this condiment.

## Figures and Tables

**Table 1:** Survey results.

Parameters	Name of markets					
	Soba market	Sinistré market	Major market	Haoussabougou market	Petit Paris market	Average
Soumbara type sold						
Granulated	100%	100%	85.7%	100%	100%	97.14%
Powder	80%	100%	71.4%	60%	100%	82.28%
Paste	0%	0%	85.7%	60%	25%	34.14%
Type requested						
Granulated	60%	0%	28.6%	60%	25%	34.72%
Powder	40%	100%	57.1%	40%	75%	62.42%
Paste	0%	0%	14.3%	0%	0%	2.86%
Supplying						
Producer	100%	100%	100%	100%	100%	100%
Sale						
More than one day	100%	100%	100%	100%	100%	100%
Storage unsold Soumbara						
Store	100%	100%	100%	100%	100%	100%
Protection during the sale						
No protection	100%	100%	100%	100%	100%	100%

**Table 2:** pH of the different types of “Soumbara.”

Market	pH		
	Granulated	Powder	Paste
GM	6.56 ± 0.28^a^	6.28 ± 0.17^a^	6.7 ± 0.4^a^
MH	6.8 ± 0.03^a^	6.05 ± 0.38^a^	6.7 ± 0.43^a^
MPP	6.8 ± 0.25^a^	6.3 ± 0.18^a^	6.6 ± 0.52^a^

In row and column, the averages affected by the same letter are not significantly different at the 5% threshold according to the Newmann-Keuls test. MH: Haoussabougou market; MPP: Petit Paris market; MG: Major market.

**Table 3:** Titratable acidity of the different types of “Soumbara.”

Market	Titratable acidity (% acidity)		
	Granulated	Powder	Paste
MG	0.13 ± 0.12^a^	0.3 ± 0.08^b^	0.1 ± 0.02^a^
MH	0.1 ± 0.01^a^	0.25 ± 0.15^ab^	0.1 ± 0.08^a^
MPP	0.07 ± 0.01^a^	0.2 ± 0.06^ab^	0.08 ± 0.005^a^

In row and column, the averages affected by the same letter are not significantly different at the 5% threshold according to the Newmann-Keuls test. MH: Haoussabougou market; MPP: Petit Paris market; MG: Major market.

**Table 4:** Moisture content of the different types of “Soumbara.”

Market	Moisture content (%)		
	Granulated	Powder	Paste
MG	8.6 ± 1.15^a^	9.3 ± 3.05^a^	24.7 ± 3.05^b^
MH	10.7 ± 2.3^a^	7.3 ± 1.15^a^	22 ± 8.71^b^
MPP	10.7 ± 3.05^a^	9.3 ± 2.3^a^	20 ± 3.46^b^

In row and column, the averages affected by the same letter are not significantly different at the 5% threshold according to the Newmann-Keuls test. MH: Haoussabougou market; MPP: Petit Paris market; MG: Major market.

**Table 5:** Loads of mesophilic aerobic germ in the different types of “Soumbara.”

Market	Mesophilic aerobic germ loads (log_10_ cfu/g)		
	Granulated	Powder	Paste
MG	8.38 ± 1.6^a^	7.74 ± 1.19^a^	8.47 ± 1.6^a^
MH	7.83 ± 0.35^a^	6.94 ± 0.82^a^	6.76 ± 0.5^a^
MPP	6.41 ± 0.45^a^	7.47 ± 0.76^a^	6.17 ± 0.9^a^
Limit (*M*)	**6**	**6**	**6**

In row and column, the averages affected by the same letter are not significantly different at the 5% threshold according to the Newmann-Keuls test. MH: Haoussabougou market; MPP: Petit Paris market; MG: Major market.

**Table 6:** Loads of total coliforms in the different types of “Soumbara.”

Market	Total coliforms loads (log_10_ cfu/g)		
	Granulated	Powder	Paste
MG	1.71 ± 1.54^a^	2.47 ± 0.89^a^	1.64 ± 2.2^a^
MH	1.76 ± 2.11^a^	1.86 ± 0.57^a^	1.26 ± 0.47^a^
MPP	2.25 ± 2.26^a^	2.96 ± 0.68^a^	1.13 ± 1.06^a^
Limit (*M*)	3	3	3

In row and column, the averages affected by the same letter are not significantly different at the 5% threshold according to the Newmann-Keuls test. MH: Haoussabougou market; MPP: Petit Paris market; MG: Major market.

**Table 7:** Loads of mould in the different types of “Soumbara”.

Market	Mould loads (log_10_ cfu/g)		
	Granulated	Powder	Paste
MG	2.4 ± 0.69^a^	2.25 ± 0.27^a^	1.56 ± 0.51^a^
MH	1.2 ± 1.15^a^	1.78 ± 1, 58^a^	2.52 ± 0.77^a^
MPP	1.44 ± 0.42^a^	1.76 ± 0.4^a^	0.86 ± 0.8^a^
Limit (*M*)	4	4	4

In row and column, the averages affected by the same letter are not significantly different at the 5% threshold according to the Newmann-Keuls test. MH: Haoussabougou market; MPP: Petit Paris market; MG: Major market.

**Table 8:** Loads of yeast in the different types of “Soumbara.”

Market	Yeast loads (log_10_ cfu/g)		
	Granulated	Powder	Paste
MG	<1	<1	<1
MH	0.33 ± 0.57^a^	0.56 ± 0.98^a^	<1
MPP	1.15 ± 0.27^a^	1.53 ± 0.5^a^	0.75 ± 1.3^a^
Limit (*M*)	4	4	4

In row and column, the averages affected by the same letter are not significantly different at the 5% threshold according to the Newmann-Keuls test. MH: Haoussabougou market; MPP: Petit Paris market; MG: Major market.

## Data Availability

Data used to support the findings of this study are included within the article.
